# Atlantic Forest Malaria: A Review of More than 20 Years of Epidemiological Investigation

**DOI:** 10.3390/microorganisms9010132

**Published:** 2021-01-08

**Authors:** Julyana Cerqueira Buery, Filomena Euridice Carvalho de Alencar, Ana Maria Ribeiro de Castro Duarte, Ana Carolina Loss, Creuza Rachel Vicente, Lucas Mendes Ferreira, Blima Fux, Márcia Melo Medeiros, Pedro Cravo, Ana Paula Arez, Crispim Cerutti Junior

**Affiliations:** 1 Unidade de Medicina Tropical, Universidade Federal do Espírito Santo, Vitória 29047-105, Brazil; tomenaalencar@hotmail.com (F.E.C.d.A.); vicentecrachel@gmail.com (C.R.V.); lmendesf1@gmail.com (L.M.F.); blimafux@yahoo.com.br (B.F.); fil.cris@terra.com.br (C.C.J.); 2 Global Health and Tropical Medicine, Instituto de Higiene e Medicina Tropical, Universidade NOVA de Lisboa, 1349-008 Lisboa, Portugal; marcia.medeiros@ihmt.unl.pt (M.M.M.); PCravo@ihmt.unl.pt (P.C.); aparez@ihmt.unl.pt (A.P.A.); 3 Instituto de Medicina Tropical de São Paulo, Universidade de São Paulo, São Paulo 05403-000, Brazil; amrcd2@gmail.com; 4 Superintendência de Controle de Endemias do Estado de São Paulo, São Paulo 01027-000, Brazil; 5 Instituto Nacional da Mata Atlântica, Santa Teresa 29650-000, Brazil; carol.loss@gmail.com

**Keywords:** malaria, molecular epidemiology, *Anopheles*, *Plasmodium*, DNA, mitochondrial, sequence analysis, DNA, zoonoses

## Abstract

In the south and southeast regions of Brazil, cases of malaria occur outside the endemic Amazon region near the Atlantic Forest in some coastal states, where *Plasmodium vivax* is the recognized parasite. Characteristics of cases and vectors, especially *Anopheles (Kerteszia) cruzii*, raise the hypothesis of a zoonosis with simians as reservoirs. The present review aims to report on investigations of the disease over a 23-year period. Two main sources have provided epidemiological data: the behavior of *Anopheles* vectors and the genetic and immunological aspects of *Plasmodium* spp. obtained from humans, *Alouatta* simians, and *Anopheles* spp. mosquitoes. *Anopheles (K.) cruzii* is the most captured species in the forest canopy and is the recognized vector. The similarity between *P. vivax* and *Plasmodium simium* and that between *Plasmodium malariae* and *Plasmodium brasilianum* shared between simian and human hosts and the involvement of the same vector in the transmission to both hosts suggest interspecies transfer of the parasites. Finally, recent evidence points to the presence of *Plasmodium falciparum* in a silent cycle, detected only by molecular methods in asymptomatic individuals and *An. (K.) cruzii*. In the context of malaria elimination, it is paramount to assemble data about transmission in such non-endemic low-incidence areas.

## 1. Introduction

**Figure 1:**
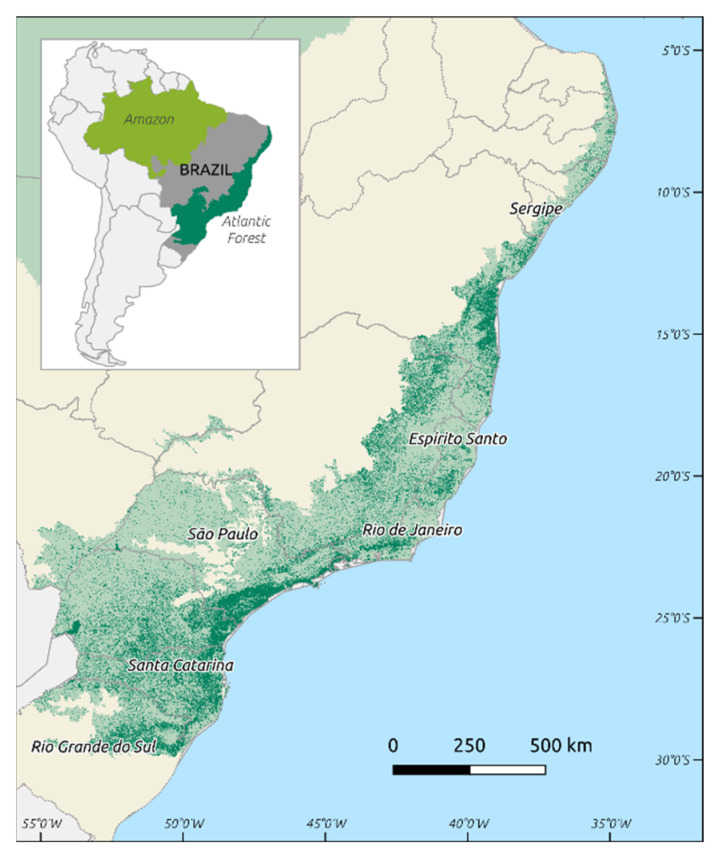
Map of Brazil, with the Brazilian Amazon in light green and the Brazilian Atlantic Forest in dark green: darker areas in the Atlantic Forest correspond to remaining forest fragments [7].

*Plasmodium vivax*, *Plasmodium falciparum*, and *Plasmodium malariae* are the most common etiologic agents of human malaria in the Americas. Brazil registered 194,271 cases in 2018 [1], placing the country in second place in a list of malaria frequency in these regions [1]. Cases of *P. vivax* infection are the most common, and the Amazonian region is the most affected area in the country, with 99% of occurrences. The cases originating from transmission in the extra-Amazonian regions of Brazil constitute only 1%, with discrete clinical presentations reported every year and a particular transmission cycle [2,3]. Efforts to control malaria until the 1960s were focused in urban areas, and these measures left behind the dense Atlantic Forest (Figure 1) that was neglected by health authorities. Bromeliad-malaria is the name of the disease in such areas because the mosquito vectors use the collection of water in the axils of Bromeliaceae plants to reproduce [4]. Endemic areas are relatively frequent in the rural communities located in the coast of southeastern Brazil, and *P. vivax* is the parasite responsible for human infections. However, it is important to stress that there are also records of *P. malariae* and *P. falciparum* infections, albeit in a lower proportion [2,3,5,6].

Bromeliad-malaria is transmitted in the Atlantic Forest system by mosquitoes of the *Kerteszia* subgenus, with *Anopheles (Kerteszia) cruzii* being the most abundant [8,9,10,11,12]. Infections can occur in hosts that are blood meal sources for the mosquitoes generally located on the top of trees because of the acrodendrophilic behavior of these insects (the tendency of certain wild species to live and preferentially feed in the canopy). When these mosquitoes come down to lower heights, they feed on non-usual hosts such as humans. The acrodendrophilic behavior of *An. (K.) cruzii* is well documented, but several studies have shown that this species may also occur in high densities at ground level in the forest, including infected specimens [13,14].

**Figure 2:**
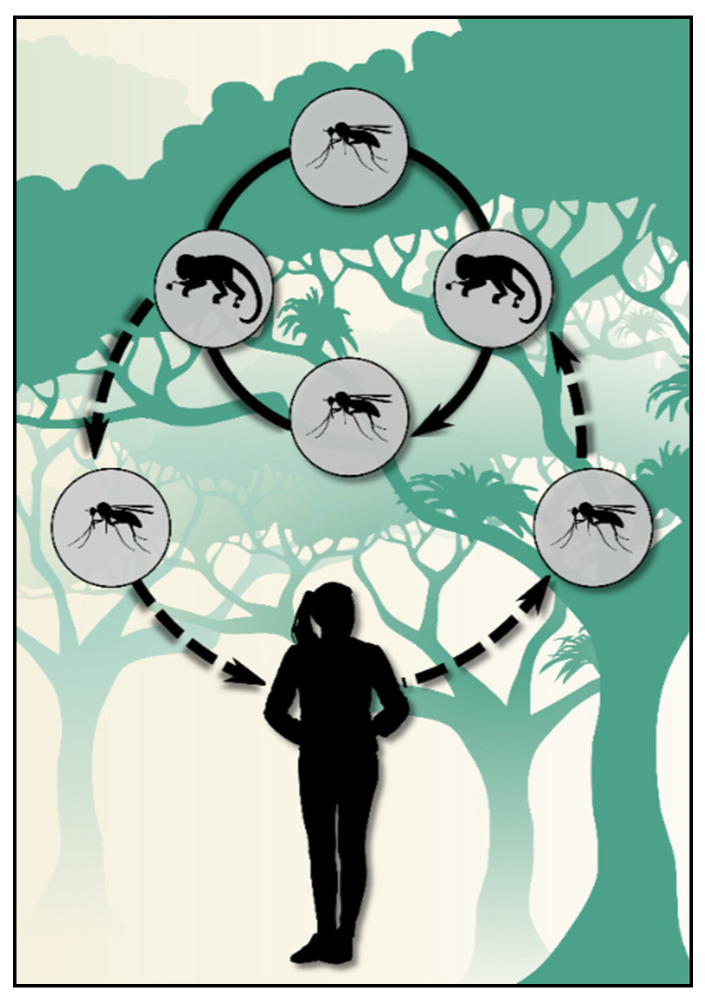
Scheme of the transmission cycle of bromeliad-malaria that is hypothesized at the Atlantic Forest biome.

The transmission cycle in this region does not fit the traditional malaria cycle, as cases occur at large distances from each other and are often separated by several weeks. The existence of a zoonosis, with infected simians participating in the epidemiology, is hypothesized (Figure 2). Despite *Plasmodium simium* and *Plasmodium brasilianum* being implicated as the etiologic agents of simian infections, several studies have suggested that *P. vivax* and *P. malariae* are the same species as *P. simium* and *P. brasilianum*, respectively, based on their genetic and morphological similarities [15,16,17,18,19].

The problem of malaria zoonotic transmission was first seriously considered regarding *Plasmodium knowlesi* in Southeast Asia [20]. It was again reinforced when a natural transmission of *Plasmodium cynomolgi* became known in 2011 in Malaysia [21]. If the *sustainable development goals* are to be achieved concerning malaria elimination, such areas of zoonotic transmission should be addressed properly as they are not responsive to standard control measures, precluding achievement of the goals by 2030.

Additionally, the ecosystem balance, involving sustainable use of natural resources and preservation of ecologic niches, is necessary to preserve the well-being of populations under the One Health concept. Therefore, any control activity aiming to interrupt a zoonotic transmission should not interfere with the abovementioned balance, especially in a country where the disease is a significant public health problem.

Several studies have been conducted by different groups from the Brazilian states affected by this form of autochthonous malaria. Some consider the disease a focal zoonosis that occasionally appears, for example, in tourists that visit the forest for leisure and end up being infected by simian parasites [22,23]. However, in other settings, bromeliad-malaria continues to be reported in native inhabitants of rural cities every year, often in people that have never left the areas where they were born but exert some kind of professional activity inside the forest [2,3,5]. These differences between the transmission sites are being better understood now as epidemiological studies have used molecular tools to differentiate the parasites found in the three *Plasmodium* hosts (mosquitoes, simians, and humans) in these regions of Brazil [5,24,25,26].

Although a lot of knowledge has already been gathered, new questions have also been raised. This review intends to shed light upon the main aspects of bromeliad-malaria transmission, based on studies conducted using classical epidemiology, molecular epidemiology, and seroepidemiology since 1997.

## 2. Simian *Plasmodia* and Their Relationship with Human Malaria in the Atlantic Forest

The hypothesis that wild monkeys in the Atlantic Forest play an important role in the areas of residual malaria transmission has been suggested for many decades [27,28,29,30,31,32]. The existence of monkeys infected with *P. brasilianum* and *P. simium*, similar to the human parasites *P. malariae* and *P. vivax*, respectively, raises the possibility of a zoonosis occurrence in this biome, as suggested in some studies mainly involving howler monkeys (*Alouatta guariba clamitans*), the ones most frequently found infected [3,29,32,33].

Natural infection by *P. brasilianum* has been described in approximately 31 species of New World monkeys from Costa Rica to Brazil [25,29,34,35]. Additionally, some phylogenetic studies suggest that *P. brasilianum* represents an anthropozoonosis acquired by New World monkeys from humans who migrated from Africa [35,36,37,38].

Natural infection by *P. simium* has been described in howlers monkeys from Atlantic Forest, in *Alouatta* genera (Atelidae family) by Fonseca (1951), later in woolly spider monkeys (*Brachyteles arachnoides*) [29,39], and more recently in black-fronted titi monkey *Callicebus nigrifrons* (Pitheciidae family) [40] and the capuchin monkeys *Sapajus xanthosternos* and *Sapajus robutus* (Cebidae family) [41].

Regarding the zoonotic infection, the first human case described in Brazil occurred with a park ranger who participated in entomological collections in Serra da Cantareira, São Paulo municipality, and developed malaria by collecting mosquitoes in the canopy [42]. *Plasmodium simium* was then identified as the etiologic agent, and *An. (K.) cruzii* was identified as the vector. The clinical manifestations were mild and resolved spontaneously.

Since *P. vivax* is the main etiological agent in the Atlantic Forest, understanding the phylogenetic relationship between *P. vivax* and *P. simium* and their past population dynamics is crucial for characterization of the zoonotic picture. Genetic similarities between *P. simium* and *P. vivax* were reported in studies using sequences of genes such as the circumsporozoite protein (CSP) and merozoite surface protein 1 (*msp-1*), mitochondrial cytochrome b (*cytb*), and microsatellite molecular markers [15,16,17,18,19]. In addition, Costa et al. (2015) [43] conducted research with *P. simium* in southern Brazil, in the state of Santa Catarina, demonstrating recent transfer from human plasmodia to monkeys of the New World based on the genetic diversity found in the Duffy Binding Protein gene (PvDBP) sequences of these animals. Studies carried out with nuclear genes, CSP and 18S small ribosomal subunit (18S rRNA), as well as mitochondrial genes *cytb* and cytochrome c oxidase subunit I (*cox*) indicate a recent expansion of *P. vivax* [19]. They also demonstrate that genetic divergence within *P. vivax* is greater than genetic divergence between *P. simium* and some strains of *P. vivax*, suggesting a recent transfer between the two hosts [19].

Recent research suggested parasite transfer between monkeys and humans in the Atlantic Forest of Rio de Janeiro. Brasil et al. (2018) [22] and Alvarenga et al. (2018) [25] demonstrated that common haplotypes in parasite mitochondrial DNA in howler monkeys and human patients indicate that malaria behaves as an anthropozoonosis. They also proposed that these haplotypes could be used as markers to identify *P. simium* infection in humans. The same authors conducted a complementary study with samples of howler monkeys from Rio de Janeiro and identified a few single nucleotide polymorphism (SNPSs) that were suggested to be useful to distinguish *P. simium* from *P. vivax* [23].

However, other studies have shown the predominance of a typical *P. vivax* haplotype from the Amazon region in humans, simians, and anophelines in the Atlantic Forest and revealed the genetic diversity of P. simium/P. vivax* circulating in simian hosts, suggesting that identification of only a few SNPs may be not enough to distinguish these parasites [5,24].

A mitogenome analysis performed by Rodrigues et al. (2018) [24] strongly indicated the transfer of *P. vivax* from humans to monkeys and a low diversity of *P. simium* strains when compared to *P. vivax* strains from the Atlantic Forest and Amazon. However, it will be necessary to aggregate more data from wild primates, including other species, to infer the distribution of different strains in the Atlantic Forest. Altogether, the sum of evidence from the several studies cited suggests that *P. simium* and *P. vivax* are the same species.

Recently, Mourier et al. (2020) analyzed the complete genome of *P. simium*, showing that it is monophyletic and nested within the wider diversity of South American *P. vivax*. Interestingly, the same study also reported significant differences between *P. vivax* and *P. simium* in the genes encoding region 1 of the Duffy binding protein 1 (DBP1) and the reticulocyte binding protein 2 (RBP2a). The authors suggest that these changes in the key encoding erythrocyte invasion ligands together with other genetic changes possibly facilitated the transfer of *P. simium* to humans [44].

In recent years, studies of simian malaria have focused on the detection of *Plasmodium* in blood samples through an invasive process often harmful to wild specimens, usually causing injury or death during containment and anesthesia. Nowadays, alternative noninvasive methodologies have enabled safer and more effective screening, allowing the detection of parasite DNA using other types of samples, such as feces, urine, and saliva [38,45,46,47,48,49,50,51,52].

Diagnostic methods for simian and human malaria, through the analysis of DNA in feces, have been developed, allowing for sensitive detection of *Plasmodium* spp. and assuring that this diagnostic method is as effective as the one based on blood collection [48,49,51,53]. In Southern Brazil (state of Santa Catarina), in an area of Atlantic Forest, a study was carried out to diagnose simian malaria using fecal samples, and the results corroborated those of the previous studies in Africa and Asia [54].

Siregar et al. (2015) [49] analyzed infections in monkeys of the genus *Macaca* in Southeast Asia, in which it was possible to identify and quantify parasites (*cytb* gene). Those authors also inferred possible explanations for the presence of *Plasmodium* DNA in the feces: by passive entry into the stool via serum or into macrophage phagosomes in the host’s reticuloendothelial system or by the passage of free DNA from degraded parasites in the liver that fall into the feces through bile. Using an experimental malaria model, Abkallo et al. (2014) [55] identified rodent *Plasmodium* in the liver, bile, and feces after inoculation of sporozoites. The DNA found in the fecal material may reflect the chronicity of infections in the hosts, but it may not be related to the occurrence of the blood cycle.

## 3. Vector Behavioral and Environmental Changes as Important Factors Related to Bromeliad-Malaria Transmission

In Brazil, there is a wide variety of *Anopheles* mosquitoes. Depending on the climate, temperature, landscape, and level of urbanization, the behavior of these insects may change, which is reflected in the disease transmission. In the Amazon region, the main vectors of malaria belong to the subgenus *Nyssorhynchus*, namely *Anopheles (Nyssorhynchus) darlingi* and *Anopheles (Nyssorhynchus) albitarsis* [56,57]. However, although *Nyssorhynchus* also populates regions outside the Amazon, there are species of the subgenus *Kerteszia* that are prominent in states with cases of bromeliad-malaria [58]. The anophelines responsible for transmission of the residual form of disease belong to such a subgenus, mainly *Anopheles (Kerteszia) cruzii* but also *Anopheles (Kerteszia) bellator* and *Anopheles (Kerteszia) homunculus* [3,59,60,61].

These mosquitoes have been observed in areas of the Atlantic Forest that extend along the Atlantic coast of Brazil, from Rio Grande do Sul state in the south to Sergipe state in the northeast. The presence and abundance of these vectors are closely related to bromeliads, a native plant of the Atlantic Forest known to accumulate water in its leaf axils, providing an ideal breeding site for reproduction of these insects, especially mosquitoes of the subgenus *Kerteszia* [13].

Considered the major vector of bromeliad-malaria, *An. (K.) cruzii* appears to be the only natural vector capable of transmitting simian malaria in the southeastern and southern regions [62]. Morphological, molecular, and genetic aspects of this species have been studied over time, suggesting that *An. (K.) cruzii* belongs to a complex of cryptic species, with at least three distinct lineages occurring in different sites in the Atlantic Forest [63,64,65,66]. *Anopheles (Kerterszia) bellator* DYAR & KNAB, 1906 and *An. (K.) homunculus* KOMP, 1937 have a smaller epidemiological importance and therefore play a secondary role in the transmission of bromeliad-malaria [13].

The importance of *An. (K.) cruzii* as a natural vector of simian and human malarias in the Atlantic Forest has been evidenced in several studies. In the mountain region of the state of Espírito Santo, *An. (K.) cruzii* was found naturally infected with P. vivax/P. simium* on two occasions [8,9]. In the coastal and mountainous areas of Serra do Mar (in the state of São Paulo), this species has been found naturally infected with P. vivax/P. simium*, *P. vivax* VK247 (variant), *P. malariae*/P. brasilianum*, and more recently *P. falciparum* [6,67,68].

In autochthonous malaria transmission settings, vector behavior can help to explain some of the unconventional transmission. The presence of competent vectors, their distribution in space, and their behavior in the environment seem to be causally related to the spread of native malaria. To understand bromeliad-malaria, the vector characteristics associated with this transmission are important and should be debated.

Acrodendrophilia, a behavioral characteristic of mosquitoes of the subgenus *Kerteszia*, especially *An. (K.) cruzii*, refers to the preference of this vector to practice hematophagy in the canopy, occasionally descending to the ground, resulting in feedings in both strata of the forest [13]. According to Consoli and Lourenço-de-Oliveira (1994), this preference is explained by the fact that these mosquitoes reproduce inside bromeliads located under the shade of the tree tops, protecting them from the sun’s rays and avoiding evaporation of the water contained inside the plants [13]. Rezende and colleagues (2009), in a study conducted between 2004 and 2005 in the mountainous region of Espírito Santo, where they systematically captured anophelines in various forest environments, found the greatest diversity of species in the peridomicile [9]. *Anopheles (K.) cruzii* was captured exclusively in the interior of the forest and was markedly acrodendrophilic, since 90.8% of the specimens were captured in the treetops. In a second instance, in the same region, the authors demonstrated again the acrodendrophilia of *An. (K.) cruzii*, which was more pronounced inside the forest [10]. The ratio between specimens captured in the canopy and in the ground was 799/4 in the interior of the forest and 142/29 in its fringe [9]. Ten years later, Buery and colleagues (2018) returned to this area and updated the information about the vector behavior, and *An. (K.) cruzii* still prevailed as the main vector found at the trapping station, with 99.05% of specimens being captured in the canopy [8].

Studies have confirmed this acrodendrophilic characteristic of *An. (K.) cruzii*, but variations in its behavior were observed in areas of simian and human malaria transmission [62,69]. In environments where this insect is definitely acrodendrophilic, there are significant rates of infected monkeys and absence or rare cases of human malaria. On the other hand, in areas where it behaves in a more versatile way, moving from the canopy to ground levels, a greater number of human cases occurs. This vertical dispersion allows the species to feed on both nonhuman primates (NHPs) and humans, supporting the sharing of *Plasmodium* spp. among the vertebrate hosts.

Representatives of the *Kerteszia* subgenus are also known by their non-anthropic behavior, i.e., they are practically absent in the anthropic environment and their presence close to human dwellings is strictly dependent on their blood sucking behavior under exceptional conditions, usually caused by the invasion of humans into the wild. In a study by Guimarães et al. (2000) [70], *An. (K.) cruzii* was found near or inside houses performing hematophagia, which, according to the researchers, showed some attraction of the species to humans. Forattini et al. (1990) [14] described the capacity of *An. (K.) cruzii* to migrate alternately between the wild environment and the peridomicile. Furthermore, according to Guimarães et al. (2000) [70], despite discrete anthropophilia, *An. (K.) cruzii* is fundamentally associated with the wild environment since it does not remain indoors after the blood meal. The same author had already observed these characteristics earlier, corroborating what had been previously established by Forattini et al. (1993) [71]. Thus, the presence of *An. (K.) cruzii* in households only occurs due to the proximity between the dwellings and the wild environment [14,70,71].

The opportunistic behavior of *An. (K.) cruzii* is associated with its acrodendrophilia and vertical dispersion [69,72,73,74], acting as a bridge for transmission of plasmodia between monkeys and humans. Additionally, it was observed that the abundance of *An. (K.) cruzii* is related to the gradient of forest cover. In degraded environments and with a larger human presence, rarefaction of this species occurs [75]. However, even so, it was possible to find specimens infected with plasmodia in these places, reinforcing the idea of a zoonotic interaction between human and simian cycles [68].

Although *P. falciparum* is not considered an etiologic agent of malaria in the Atlantic Forest, there is some evidence suggesting that it circulates in this biome. Laporta et al. (2015) recovered DNA of this parasite from anophelines of the subgenera *Kerteszia* and *Nyssorrhynchus* collected in seven areas with different types of vegetation in the Atlantic Forest in Vale do Ribeira, in the state of São Paulo [6]. This, together with the previous finding of *P. falciparum* DNA in blood from donors living in some areas of the Atlantic Forest, underscores the need for further research to investigate the hypothesis of asymptomatic infections by this parasite or a related agent in the Atlantic Forest [76]. In addition, blood samples from humans and simians collected in field surveys in the Atlantic Forest have suggested the presence of *P. falciparum* DNA in the past [2,3,77].

Recent studies suggest that anthropogenic changes in the Atlantic Forest environment can modulate the genetic, ecological, and behavioral aspects of *An. (K.) cruzii*. Medeiros-Sousa and colleagues (2019) hypothesized that the acrodendrophilic behavior of *An. (K.) cruzii* may be influenced by microclimatic conditions such as a reduction in humidity, which is directly affected by environmental changes (e.g., deforestation) [74]. According to Multini et al. (2020), the genetic structure among the populations of *An. (K.) cruzii* collected on the ground and in the canopy of trees in the urban environment near the dense Atlantic Forest in São Paulo and the greater genetic diversity in the urban ground-level population indicates an increased insect–human contact [78].

Anthropogenic modifications of the natural habitat of mosquitoes point to a consequent shift to the anthropophilic behavior of *An. (K.) cruzii* becoming increasingly common [78,79]. Understanding the impact of human modifications in natural habitats of *An. (K.) cruzii* and other anophelines responsible for disease transmission is crucial so that health authorities may develop assertive strategies for the elimination of malaria in the Brazilian Atlantic Forest.

## 4. Human Malaria

As previously mentioned, most of the malaria transmission in Brazil occurs in the Amazon region, comprising 99% of the total burden of the disease in the country. However, the minor participation of the Residual Malaria of Atlantic Forest Systems (RMAFS) in the total of cases is of importance due to its unusual ecology. Based on the accumulated knowledge to the present day, the overall consensus is that control for this type of malaria must not follow the same path as those actions planned for the traditional transmission cycle as it is highly unlikely that it could be controlled by a strategy based on indoor spraying or a track-and-trace approach.

The initial evidence of a different cycle of malaria transmission in areas of the Atlantic Forest came from a cluster of cases occurring in workers during railroad construction in the mountains of the São Paulo state in 1898 [80,81]. The sanitary authorities in Brazil had always dealt with malaria in lowland areas, where the recognized presence of *An. (N.) darlingi* seemed to be a prerequisite for the occurrence of human cases. However, under this scenario, the landscape was mountainous and the usually incriminated vector was absent. The sharp observation of the scientist Adolpho Lutz lead to the recognition of a different transmission cycle based on the breeding of a newly identified anopheline species, later called *An. (K.) cruzii*, in the bromeliads located at the canopy [4,80,81]. Given that malaria was a public health threat along the entire country in the first half of the twentieth century, the so-called “bromeliad-malaria” did not attract much attention from those involved in the fight against the disease. Considering the immense burden imposed by several millions of cases and hundreds of thousands of deaths, the RMAFS did not seem to be more than a curiosity, given its low occurrence and benign evolution [58]. RMAFS remained forgotten during the campaigns implemented along the sixties and the seventies, except for the intense activities of deforestation and removal of bromeliads from the forest in Santa Catarina state specifically designed to put an end to such type of transmission [58,73]. The success of the twentieth century campaigns in Brazil, motivated by the eradication effort promoted by the World Health Organization at that time, resulted in virtual elimination of malaria in the country, except for the Amazon region [82,83]. However, human cases in the forested areas in the south and southeast of the country continued to be reported.

The existence and the possible importance of RMAFS were considered again only in the early nineties through the work of Curado et al. [27] in the forested areas of São Paulo state. Based on the occurrence of 33 cases over two years in two districts, the authors detected IgG and IgM antibodies against *Plasmodium* antigens in blood samples collected from 277 individuals living in a varying distance from five to ten kilometers from case dwellings, which rarely inform on the typical malaria symptoms. The seropositive rate achieved 73% as the highest frequency. Those dwellers included either permanent residents or individuals from the city that used the countryside for rest on the weekends. The authors concluded that there was malaria transmission in that biome and that further studies would be necessary [27]. Again, in 2006, Curado et al. investigated two different areas of the same biome, collecting blood from 318 individuals [77]. Three individuals had IgM antibodies against *P. vivax*, and 18 individuals had antibodies against *P. malariae*. At the two sites, the percentage of IgG-positive individuals varied from 32% to 49% for *P. vivax* and from 16% to 19.3% for *P. malariae*. Unexpectedly, PCR-amplified fragments of *Plasmodium* DNA extracted from the blood samples were not only from *P. vivax* and *P. malariae* but also from *P. falciparum* parasites. One individual harbored the DNA of *P. malariae*, two of them had that of *P. falciparum*, and three presented a mixture of *P. vivax* and *P. falciparum* [77]. The latter was an unexpected finding, as it had neither been related to clinical cases nor been morphologically identified in the Atlantic Forest systems before.

In 2007, Cerutti et al. added more information about the RMAFS characteristics [2]. The authors collected blood from 65 individuals diagnosed as malaria cases from 2001 to 2004 in Espírito Santo state. All of them had positive thick blood smears for *P. vivax*. Successful PCR-amplification of a DNA fragment from the 18S small subunit (18S rRNA) gene extracted from 48 of the 65 samples disclosed *P. vivax* in 47 and *P. malariae* in the remaining [2].

Blood collection from the inhabitants living in a two-kilometer radius around the cases’ dwellings revealed a prevalence of IgM and IgG antibodies at 15.8% and 44.6% for *P. malariae* in 253 samples, 6.2% and 37.7% for *P. vivax* in 1701 samples, and 13.5% and 13% for *P. falciparum* in 192 samples, respectively [2].

PCR for the amplification of the 18S rRNA gene performed in 1527 samples collected from the dwellers disclosed silent infection for *P. vivax* in 23 samples, for *P. malariae* in 15 samples, for *P. falciparum* in 9 samples, and a mix of *P. malariae* and *P. falciparum* in one sample, totaling a prevalence of 3.1%. In that investigation, clinical cases predominated in men (78.5%), which may be related to the different working habits and higher exposure to infective mosquito bites. Such a characteristic coupled with the fact that recognized vectors were absent either from inside or near the houses, indicated, according to the authors, a probable extra-domiciliary transmission of the infectious agents [2]. Brasil et al. later confirmed most of these characteristics in Rio de Janeiro state, except for the profile of those affected tourists entering the forest, as opposed to the agriculture workers identified by Cerutti et al. [2,22].

De Alencar et al. further explored the occurrence of asymptomatic infections in the scenario of RMAFS in reports of 2017 and 2018 [84,85], reassessed part of the positive dwellers previously identified by Cerutti et al., and followed another cohort of residents for two years in the same region. Of the 37 carriers from the 48 detected between 2001 and 2004, PCR screening based on 18S rRNA gene amplification disclosed DNA of *P. malariae* in two, the same agent identified in their samples from 2001–2004 [84]. On the other hand, in the cohort of 92 individuals newly engaged in the two year follow-up, PCR revealed a prevalence of 3.4% of asymptomatic infections, with 2.3% for each one, i.e., *P. vivax* and *P. malariae*. During the two years, the authors found an incidence of 2.5 infections for 100 person-years or 1.25 infections for 100 person-years for each species. Additionally, de Alencar et al. observed that the positive individuals spontaneously reverted to a negative state without any therapeutic intervention. Mathematical modelling based on the frequency of asymptomatic infections and the vector density suggested that the transmission cycle could not be maintained only by the human reservoir in the region [85]. Miguel et al. added evidence to the case of asymptomatic infections through their survey in the Guarapimirim municipality in Rio de Janeiro state as they also detected a prevalence of 2.8%, reporting an association between incursions to the forest environment and the probability of being infected [86].

In summary, studies on RMAFS suggest that *Plasmodium* infection occurs mainly outdoors, originating from a nonhuman reservoir. Overall, symptoms of disease are mild to moderate, the cases are sparse, and there are no outbreaks. There is evidence of the involvement of *P. vivax*, *P. malariae*, and *P. falciparum* in the transmission cycle despite the absence of clinical cases caused by the latter.

## 5. Surveillance

The health facilities responsible for malaria diagnosis in the region belong to the Brazilian Unified Health System (Sistema Único de Saúde; SUS) offering free blood smear examinations for all those referred with fever. Malaria diagnosis is exclusively done by the public Brazilian Unified Health System, meaning that no other source of diagnosis exists in the private sector. As parasite density in the blood of those infected is usually low, rapid diagnostic tests (RDT) are of little help given their low sensitivity for low parasitaemia [87]. Morphological diagnosis through thick smears by health facilities always report infections as being *P. vivax* despite the high possibility that part of these cases are caused by *P. malariae*, instead. The epidemiological investigations performed in the affected areas used several molecular techniques, mainly PCR directed to the 18S rRNA gene, but also different techniques of DNA sequencing [5,26].

Recently, mitochondrial DNA sequencing raised several debates about putative evidence of a zoonotic cycle [5,22,23,24,25,26]. When comparing sequences generated by the sequencing systems with those deposited in GenBank and analyzing phylogenetic trees, some studies suggested a transfer of the parasites from the human host to nonhuman primates but not as a fully recognizable zoonosis. The genetic diversity of the parasites recovered from humans is higher than the diversity of parasites from nonhuman reservoirs, which may indicate a primary transfer from the humans to the simians [5,24].

Altogether, the only certainty to date is that malaria occurs in the Atlantic Forest systems as a process of transference between primate species (human and nonhumans). There is a movement of *spillover* and *spillback* for which intensity and direction are yet impossible to be fully characterized [Duarte et al., paper in press].

## 6. Concluding Remarks

A world without malaria has been the purpose of health authorities and researchers for a long time, all of them approaching the problem from the perspective of a transmission cycle encompassing mosquitoes and human beings. Consequently, these two components of the transmission became the primary targets of international efforts for malaria control. Once elimination would become a reality, spots of the disease sustained by unusual variables, as non-human reservoirs, can become sanctuaries of transmission. Such residual areas may act as a source to reintroduce the disease in the long term. That is why it is necessary to understand all the elements involved in those scenarios of unusual transmission and to design specific strategies for their containment. To tackle a complex disease like malaria, it is of utmost importance not to underestimate its capacity to resist elimination strategies.

## 7. Future Perspectives

Several aspects that represent unanswered questions regarding the transmission cycle of RMAFS must be further explored. More studies should clarify the role of *P. falciparum* in asymptomatic infections in this extra-Amazonian scenario. Additionally, the sustainability of transmission in the absence of the NHP reservoir deserves to be better investigated, taking the opportunity brought by the recent yellow fever epizootic (responsible for the death of approximately 90% of the *Alouatta* specimens in the biome). Finally, improved understanding of the genetic structure of the parasite species can foster our capability to develop preventive measures, such as biologic control strategies or vaccines. A world without malaria is possible, but its achievement will require the broadest understanding possible of several peculiarities of this complex infectious disease.

* The description of the species separated by “/” in this text means a lack of clarity regarding their status, i.e., both names may be designations of the same species.

## Data Availability

No new data were created or analyzed in this study. Data sharing is not applicable to this article.
